# Literary Notes

**Published:** 1900-11

**Authors:** 


### LITERARY NOTES.

THE New Zealand *Medical Journal* is a new candidate for professional favor. The first number bears date August, 1900, and is an excellent specimen of British Colonial journalism, both as to material and mechanical construction. It is edited by Dr. J. Malcom Mason, and is published for the New Zealand branch of the British Medical Association. It is printed by John Mackey, government printer, at Wellington, N.Z.

---

A New Therapeutic Reference for Physicians and Students has been published recently by W. R. Warner & Co., of Philadelphia. It is a duodecimo volume of 224 pages and contains many excellent tables of value to both physicians and students, besides much therapeutic material that will more than repay its cost. It can be had upon payment of 25 cents, bound in cloth, or for 50 cents, in flexible leather. Address the publishers, W. R. Warner & Co., 639 North Broad Street, Philadelphia, Pa.

---

MESSRS. BATTLE & CO., of St. Louis, are sending out a series of twelve pamphlets, each to contain an anatomical drawing in colors, illustrating fractures of the long bones. The drawing for September was a fracture of the femur below the trochanters, with muscles colored in red. It is artistic as well as anatomically correct. The series will be sent to any physician who will apply to Messrs. Battle & Co., 2001 Locust St., Louis, Mo.

---

THE New York Post-Graduate Medical School and Hospital has issued its 19th Annual Announcement. The largest number of physicians in the history of the school attended last year—namely, 609. They came from 43 different states, territories and countries. This is easily the most famous post-graduate medical school in America.

